# Supplementary material for: The crystal structure of bromide-bound GtACR1 reveals a pre-activated state in the transmembrane anion tunnel
Source: eLife. 2021 May 17;10:e65903. doi: 10.7554/eLife.65903 (PMC8172240; doi:10.7554/eLife.65903)
Supplement: Transparent reporting form [file elife-65903-transrepform.docx]

***eLife’s* transparent reporting form**

We encourage authors to provide detailed information *within their submission* to facilitate the interpretation and replication of experiments. Authors can upload supporting documentation to indicate the use of appropriate reporting guidelines for health-related research (see EQUATOR Network), life science research (see the BioSharing Information Resource), or the ARRIVE guidelines for reporting work involving animal research. Where applicable, authors should refer to any relevant reporting standards documents in this form.

If you have any questions, please consult our Journal Policies and/or contact us: [editorial@elifesciences.org.](mailto:editorial@elifesciences.org)

# Sample-size estimation

- You should state whether an appropriate sample size was computed when the study was being designed
- You should state the statistical method of sample size computation and any required assumptions
- If no explicit power analysis was used, you should describe how you decided what sample (replicate) size (number) to use

Please outline where this information can be found within the submission (e.g., sections or figure legends), or explain why this information doesn’t apply to your submission:

A statement about a sample size determination is included in the Methods section: “Sample size was estimated from previous experience and published work on a similar subject, as recommended by the NIH guidelines.”

# Replicates

- You should report how often each experiment was performed
- You should include a definition of biological versus technical replication
- The data obtained should be provided and sufficient information should be provided to indicate the number of independent biological and/or technical replicates
- If you encountered any outliers, you should describe how these were handled
- Criteria for exclusion/inclusion of data should be clearly stated
- High-throughput sequence data should be uploaded before submission, with a private link for reviewers provided (these are available from both GEO and ArrayExpress)

Please outline where this information can be found within the submission (e.g., sections or figure legends), or explain why this information doesn’t apply to your submission:

# The information about replicates appears in the Methods section as following: “Individual transfected HEK293 cells were selected for patching by inspecting their tag fluorescence; non-fluorescent cells were excluded, as were cells for which we could not establish a gigaohm seal. Results obtained from different individual cells were considered as biological replicates. The raw data obtained in individual cells are shown as diamonds. Sample size was estimated from previous experience and published work on a similar subject, as recommended by the NIH guidelines. No outliers were excluded.”

# Statistical reporting

- Statistical analysis methods should be described and justified
- Raw data should be presented in figures whenever informative to do so (typically when N per group is less than 10)
- For each experiment, you should identify the statistical tests used, exact values of N, definitions of center, methods of multiple test correction, and dispersion and precision measures (e.g., mean, median, SD, SEM, confidence intervals; and, for the major substantive results, a measure of effect size (e.g., Pearson's r, Cohen's d)
- Report exact p-values wherever possible alongside the summary statistics and 95% confidence intervals. These should be reported for all key questions and not only when the p-value is less than 0.05.

Please outline where this information can be found within the submission (e.g., sections or figure legends), or explain why this information doesn’t apply to your submission:

The information about statistical analysis appears in Methods section as following: “Normality of the data was not assumed, and therefore non-parametric statistical tests were used as implemented in OriginPro 2016 software; P values > 0.05 were considered not significant”, and in the Figure 2B legend: “RI > 1 by one-sample Wilcoxon signed-rank test: * p < 0.05, ** p < 0.01, n.s. not significant (p > 0.05). (B) Peak current at 60 mV. Comparison with the wild-type by Mann-Whitney test: * p < 0.005.”

(For large datasets, or papers with a very large number of statistical tests, you may upload a single table file with tests, Ns, etc., with reference to sections in the manuscript.)

# Group allocation

- Indicate how samples were allocated into experimental groups (in the case of clinical studies, please specify allocation to treatment method); if randomization was used, please also state if restricted randomization was applied
- Indicate if masking was used during group allocation, data collection and/or data analysis

Please outline where this information can be found within the submission (e.g., sections or figure legends), or explain why this information doesn’t apply to your submission:

The information about group allocation appears in Methods section as following: “Batches of culture were randomly allocated for transfection with a specific mutant; no masking (blinding) was used.”

# Additional data files (“source data”)

- We encourage you to upload relevant additional data files, such as numerical data that are represented as a graph in a figure, or as a summary table
- Where provided, these should be in the most useful format, and they can be uploaded as “Source data” files linked to a main figure or table
- Include model definition files including the full list of parameters used
- Include code used for data analysis (e.g., R, MatLab)
- Avoid stating that data files are “available upon request”

Please indicate the figures or tables for which source data files have been provided:
